# Time Trends in Disseminated Intravascular Coagulation in Japan

**DOI:** 10.31662/jmaj.2020-0071

**Published:** 2020-10-02

**Authors:** Mineo Kurokawa

**Affiliations:** 1 Department of Hematology and Oncology, Graduate School of Medicine, The University of Tokyo, Tokyo, Japan

**Keywords:** disseminated intravascular coagulation, time trends, underlying diseases, mortality, hospital stay

Disseminated intravascular coagulation (DIC) is a potentially life-threatening disease characterized by activated coagulation leading to hemorrhage due to coagulation factor consumption, and organ injury related to thrombosis-induced ischemia ^(1)^. In DIC, coagulation is inappropriately activated by various reasons that constitute underlying diseases. Super-activated coagulation often results in the acceleration of secondary fibrinolysis that is another cause of serious hemorrhage in DIC. The real aspects of clinical courses of DIC remain elusive thus far despite the efforts of many researchers. One of the reasons is that there are many factors that affect the outcomes of DIC including diagnosis criteria, underlying diseases, and choices of treatment options. DIC is usually associated with specific underlying conditions, outcomes, and optimal therapy depending on the individual patient ^(2)^. In addition, since several novel and promising treatment modalities have emerged in recent years, the choice of them including pre-existing treatments has become a complicated and challenging issue that clinicians have to face in patient care.

For decades, the Japanese government has established a novel inpatient database system named the Japanese Diagnosis Procedure Combination (DPC). By utilizing this system, we can now collect nationwide data on inpatient medical practice in the real world. This is an extremely valuable source since we can have all of the data derived from all hospitals that use DPC in Japan. Today, given that most of the principle hospitals use DPC, these data are very strong to grasp the overall tendency of the natural histories, cares, and outcomes of individual diseases in Japan.

In this issue, Yamakawa K et al. investigated time trends in underlying conditions, mortality, patient outcomes, and drug therapy preferences of DIC by analyzing DPC data ^(3)^. One of the emerging facts from their study is that the 28-day mortality has significantly decreased in recent years. This decrease is most prominent in leukemia- or sepsis-associated DIC, suggesting the advance in managements of these diseases may have contributed to outcomes of DIC. We should always keep in mind that the success of DIC treatments primarily depends on the control of the underlying disease of DIC. From this point of view, it is interesting to see whether improvements of outcomes of these two causes are superior to those of other causes of DIC.

Another point they found is that the use of thrombomodulin has significantly increased over time. To treat DIC, one has to consider the type of DIC. Typically, DIC can be divided into coagulation-dominant and fibrinolysis-dominant states, which are mainly determined by the underlying diseases. Optimal treatment varies depending on which type the DIC belongs to. Among them, thrombomodulin is an effective therapeutic modality that can be used for both types of DIC ^(4)^. It is interesting and awaits further investigation whether the use of thrombomodulin contributes to the improvement of outcomes of DIC regardless of underlying conditions.

Finally, they found that the length of hospital stay clearly decreased in these 8 years. This finding is important in terms of medical resources and finance. The costs of DIC management may be elevated due to the advents of various novel drugs. These new therapeutics should be evaluated in terms of total costs together with the decrease in hospital stay brought by effectiveness of new drugs. These kinds of investigation may be one of the most important future themes evoked by the present study.

## Article Information

### Conflicts of Interest

Mineo Kurokawa received lecture fees from Asahi Kasei Corporation.

### Disclaimer

Mineo Kurokawa is one of the Editors of JMA Journal and on the journal's Editorial Staff. He was not involved in the editorial evaluation or decision to accept this article for publication at all.
